# Editorial: Computational analysis of promoters in prokaryotic genomes

**DOI:** 10.3389/fmicb.2023.1242139

**Published:** 2023-07-10

**Authors:** Hao Lin, Yongchun Zuo, Ettayapuram Ramaprasad Azhagiya Singam

**Affiliations:** ^1^Center for Informational Biology, University of Electronic Science and Technology of China, Chengdu, China; ^2^College of Life Sciences, Inner Mongolia University, Hohhot, China; ^3^Molecular Graphics and Computation Facility, College of Chemistry, University of California, Berkeley, Berkeley, CA, United States

**Keywords:** promoter, prokaryote, artificial intelligence, sequence, prediction

Promoters are DNA sequence fragments located upstream of structural gene, which start gene transcription by combining with RNA polymerase. It has been found that in Prokaryote, promoters are considered to be key elements for Sigma factor recognition in the transcription process. By changing the promoter sequence, gene expression can be regulated. At present, enough prokaryotic promoter sequences have been accumulated, and multiple prokaryotic promoter databases have been constructed, such as PPD (Su et al., 2021), RegulonDB (Tierrafria et al., 2022), Pro54DB (Liang et al., 2017) and DBTBS (Sierro et al., 2008). The study of prokaryotic promoters will provide more useful information for understanding microbial gene transcription. This Research Topic aims to provide an important scientific communication platform for the analysis of prokaryotic promoters using artificial intelligence and big data techniques, including the development and application of computing methods and technologies for the analysis and research of prokaryotic genome promoters.

In this Research Topic, nine papers were published, five of which are about the use of artificial intelligent techniques to identify the prokaryotic promoter sequence.

Zulfiqar et al. developed a random forest (RF)-based model to predict promotors in *Agrobacterium Tumefaciens* strain C58. In the model, promotor sequences were encoded by accumulated nucleotide frequency, *k*-mer nucleotide composition, and binary encodings, and then optimized by using correlation and the mRMR-based algorithm. They inputted these optimized features into RF classifier to classify promotor sequences. The examination of 10-fold cross-validation (CV) showed that the proposed model could yield an overall accuracy of 0.837. They have also discussed the limitations and the future perspective of this study. Lin Y. et al. also developed a model to predict promotors in *Klebsiella Aerogenes*. In their model, they have utilized pseudo *k-*tuple nucleotide composition and position-correlation scoring function to encode the promotor sequences. They have also utilized mRMR to optimize the encoded features. Afterwards, they inputted the optimized features into support vector machine (SVM)-based classifier to recognize promotor sequences. Results on 10-fold CV showed the overall accuracy of 96.0%. They have also discussed about the future perspectives of this study. Li R. et al. developed a promoter prediction model for *Corynebacterium glutamicum* based on novel feature by calculating statistical parameters of multiple physicochemical properties (Li H. et al.). Feature dimensionality is effectively reduced by using variance analysis and hierarchical clustering. Finally, they achieved an accuracy of 91.6%. They briefly analyzed the importance of feature selection and validated the robustness of the model. Sumeet et al. focused on sigma70 promoter in *Escherichia coli* K-12 strains. They used over 8000-dimension features to formulate samples (Patiyal et al.). By utilizing SVM as classifier, they achieved the maximum accuracy 97.38% with AUROC 0.99 on training dataset by using 200 most relevant features. They established a webserver for using by wet-experimental scholars. Shujaat et al. designed a powerful computational model to identify phage promoters and their types (Shujaat et al.). Ten distinct feature encoding approaches were investigated in this work. Finally, a 1-D convolutional neural network model combined with one-hot encoding approach was proposed to construct model. They also built a freely web server.

Transcription factors (TFs) are important regulators for gene expression. Zheng et al. presented a capsule network-based method to identify TFs. Their model obtained an accuracy of 0.8820. They also constructed a user-friendly web server for all scientific researchers.

Bo, Sun, Ning et al.; Bo, Sun, Li et al. submitted two works for mRNA splice regulation. They first presented a novel approach to analyze the association characteristics between post-spliced introns and their corresponding mRNA based on binding free energy weighted local alignment algorithm method. They briefly introduce the advantages of binding free energy weighted local alignment algorithm method to analyze the interaction of RNA-RNA, compared with Smith-Waterman local alignment-based algorithm method. They also discussed biological significance and evolutionary mechanism of the interaction between introns and mRNAs. Subsequently, they studied the ubiquitous conservative interaction patterns between post-spliced introns and their mRNAs revealed by genome-wide interspecies comparison. They also discussed show that there are abundant functional units in the introns, and these functional units are correlated structurally with all kinds of sequences of mRNA.

Although previous studies have revealed that introns play an important role in regulating gene expression and participate in gene evolution, but the function of introns is far from clear, and are being studied from different perspectives. In the work of Li R. et al., the characteristics of the optimal matched segments between the first intron and the reverse complementary sequences of other introns of each gene were analyzed, some interesting results had been gotten. The results in this paper showed that the characteristics of the optimal matched segments presented varied regular variation along with the evolution of eukaryotes. It is found that some optimal matched segments may be related to non-coding RNA with special biological functions, just like siRNA and miRNA, they may play an important role in the process of gene expression and regulation. And perhaps the optimal matched segments with special characteristics in the first introns may take part in regulating gene expression by RNA matching competition with other introns or exon.

## Author contributions

All authors listed have made a substantial, direct, and intellectual contribution to the work and approved it for publication.

## References

[B1] Liang Z. Y. Lai H. Y. Yang H. Zhang C. J. Yang H. Wei H. H.. (2017). Pro54DB: a database for experimentally verified sigma-54 promoters. Bioinformatics 33, 467–469. 10.1093/bioinformatics/btw630 28171531

[B2] Sierro N. Makita Y. De Hoon Nakai M. (2008). DBTBS: a database of transcriptional regulation in Bacillus subtilis containing upstream intergenic conservation information. Nuc. Acids Res. 36, D93–96. 10.1093/nar/gkm910 17962296 PMC2247474

[B3] Su W. Liu M. L. Yang Y. H. Wang J. S. Li S. H. Lv H.. (2021). PPD: a manually curated database for experimentally verified prokaryotic promoters. J. Mol. Biol. 433, 166860. 10.1016/j.jmb.2021.166860 33539888

[B4] Tierrafria V. H. Rioualen C. Salgado H. Lara P. Gama-Castro S. Lally P.. (2022). RegulonDB 11, 0. Comprehensive high-throughput datasets on transcriptional regulation in Escherichia coli K-12. Microb. Genom. 8, 833. 10.1099/mgen.0.000833 35584008 PMC9465075

